# Supplementary material for: Subclass IId bacteriocins targeting mannose phosphotransferase system—Structural diversity and implications for receptor interaction and antimicrobial activity
Source: PNAS Nexus. 2024 Sep 3;3(9):pgae381. doi: 10.1093/pnasnexus/pgae381 (PMC11403280; doi:10.1093/pnasnexus/pgae381)
Supplement: pgae381_Supplementary_Data [file pgae381_supplementary_data.pdf]

Supplementary materials

Subclass IId bacteriocins targeting mannose phosphotransferase system—structural diversity and implications for receptor interaction and antimicrobial activity

ALEKSANDRA TYMOSZEWSKA AND TAMARA ALEKSANDRZAK-PIEKARCZYK*

Institute of Biochemistry and Biophysics, Polish Academy of Sciences (IBB PAS), Pawińskiego 5a, 02-106 Warsaw, Poland

*Corresponding author. Mailing address: IBB PAS, Pawińskiego 5a, 02-106 Warsaw, Poland, Phone: (+48) 22 592 1213, E-mail: tamara@ibb.waw.pl

Table S1. Bacterial strains, plasmids and primers used in this study

Strains, plasmids, primers, peptides	Description, NCBI accession number	Source* (reference)
**Strains**		
Apilactobacillus kunkeei AH1	indicator strain	LMGT NMBU
Apilactobacillus kunkeei AH38	indicator strain	LMGT NMBU
Apilactobacillus kunkeei AH119	indicator strain	LMGT NMBU
Bacillus cereus IBB3390	indicator strain	IBB PAS
Bacillus subtilis BSB1	indicator strain	(1)
Campylobacter jejuni 12	indicator strain	(2)
Campylobacter jejuni 480	indicator strain	(3)
Campylobacter jejuni 81176	indicator strain	(4)
Campylobacter coli 23/1	indicator strain	(2)
Candida albicans CAI-4	indicator strain,	(5)
Carnobacterium maltaromaticum IBB3447	indicator strain	IBB PAS
Enterococcus durans IBB3441	indicator strain	IBB PAS
Enterococcus faecalis IBB3439	indicator strain	IBB PAS
Enterococcus faecalis IBB3444	indicator strain	IBB PAS
Enterococcus faecium LMGT 2783	indicator strain	LMGT NMBU
Enterococcus faecium LMGT 2787	indicator strain	LMGT NMBU
Escherichia coli EC1000	indicator strain, host strain	(6)
Escherichia coli TG1	indicator strain	(7)
Lacticaseibacillus paracasei IBB3418	indicator strain	IBB PAS
Lacticaseibacillus paracasei IBB3425	indicator strain	IBB PAS
Lacticaseibacillus paracasei IBB3426	indicator strain	IBB PAS
Lacticaseibacillus paracasei IBB3427	indicator strain	IBB PAS
Lacticaseibacillus paracasei IBB3428	indicator strain	IBB PAS
Lacticaseibacillus paracasei LOCK 0919	indicator strain, CP005486.1	LOCK (8)
Lacticaseibacillus paracasei subsp. *paracasei* IBB3423	indicator strain, ASM973948v1	IBB PAS (9)
Lacticaseibacillus rhamnosus IBB3429	indicator strain	IBB PAS
Lacticaseibacillus rhamnosus LOCK 0900	indicator strain, CP005484.1	LOCK (10)
Lacticaseibacillus rhamnosus LOCK 0908	indicator strain, CP005485.1	LOCK (11)
Lacticaseibacillus rhamnosus GG	indicator strain, AP011548	Dicoflor (12)
Lactiplantibacillus paraplantarum IBB3438	indicator strain	IBB PAS
Lactiplantibacillus plantarum NC8	indicator strain, AGRI00000000.1	LMGT NMBU (13)
Lactiplantibacillus plantarum WCSF1	indicator strain, AL935263.2	LMGT NMBU (14)
Lactiplantibacillus plantarum IBB3433	indicator strain	IBB PAS
Lactiplantibacillus plantarum IBB3436	indicator strain	IBB PAS
Lactiplantibacillus plantarum subsp. *plantarum* IBB3434	indicator strain	IBB PAS
Lactobacillus johnsonii IBB3155	indicator strain	IBB PAS
Lactococcus garvieae IBB3403	indicator strain	IBB PAS
Lactococcus garvieae DSM 6783	indicator strain	DSMZ
Lactococcus garvieae DSM 20064	indicator strain	DSMZ

Lactococcus garvieae DSM 20385	indicator strain	DSMZ
Lactococcus garvieae DSM 20684	indicator strain, ASM244167v1	DSMZ
Lactococcus garvieae DSM 20685	indicator strain	DSMZ
Lactococcus garvieae DSM 29394	indicator strain	DSMZ
Lactococcus garvieae DSM 100577	indicator strain	DSMZ
Lactococcus garvieae IBB66	indicator strain	IBB PAS
Lactococcus garvieae IPLA 31405	indicator strain, ASM26970v1	IPLA-CSIC (15)
Lactococcus garvieae PAQ 102015-99	indicator strain, PAQ99-XT	(16)
Lactococcus lactis IBB3404	indicator strain	IBB PAS
Lactococcus lactis IBB3411	indicator strain	IBB PAS
Lactococcus lactis QU5	indicator strain	LMGT NMBU
Lactococcus. cremoris IBB3409	indicator strain	IBB PAS
Lactococcus. lactis IBB3407	indicator strain	IBB PAS
Lactococcus lactis IL1403	indicator strain, host strain, AE005176	INRA (17)
Lactococcus raffinolactis IBB91	indicator strain	IBB PAS
Leuconostoc lactis IBB3446	indicator strain	IBB PAS
Leuconostoc mesenteroides IBB3442	indicator strain	IBB PAS
Leuconostoc mesenteroides IBB3443	indicator strain	IBB PAS
Ligilactobacillus salivarius IBB3154	indicator strain	IBB PAS
Listeria monocytogenes EGD-e	indicator strain, AL591824.1	LMGT NMBU (18)
Listeria monocytogenes ATCC 43526	indicator strain	ATCC
Listeria monocytogenes ATCC 15313	indicator strain	ATCC
Listeria monocytogenes H7762	indicator strain	CDC
Pediococcus acidilacti LMGT 2002	indicator strain	LMGT NMBU
Pediococcus parvulus IBB3448	indicator strain	IBB PAS
Pediococcus pentosaceus IBB3369	indicator strain	IBB PAS
Pseudomonas aeruginosa ATCC 9027	indicator strain	ATCC
Salmonella typhimurium TT622	indicator strain	(19)
Staphylococcus aureus ATCC 6538	indicator strain	ATCC
Staphylococcus caprae DSM-20608	indicator strain	DSMZ
Staphylococcus delphini DSM-20771	indicator strain	DSMZ
Staphylococcus epidermidis DSM-20044	indicator strain	DSMZ
Staphylococcus hyicus DSM-20459	indicator strain	DSMZ
Staphylococcus intermedius DSM-20373	indicator strain	DSMZ
Staphylococcus lugdunesis DSM-4804	indicator strain	DSMZ
Staphylococcus pseudintermedius DSM-21284	indicator strain	DSMZ
Staphylococcus saprophyticus DSM-18669	indicator strain	DSMZ
Staphylococcus schleiferi DSM-6628	indicator strain	DSMZ
Streptococcus agalactiae IBB123	indicator strain	IBB PAS
Streptococcus agalactiae IBB130	indicator strain	IBB PAS
Streptococcus mitis IBB3449	indicator strain	IBB PAS
Streptococcus parauberis IBB272	indicator strain	IBB PAS
Streptococcus sobrinus IBB3450	indicator strain	IBB PAS
Lactococcus garvieae* IBB3403—spontaneous mutants*		
LG1-G32, LG34-LG38, LG40	GarAG3^{r}; Man^{+}	This study
LGA2, LGA3, LGA6, LGA10	GarA^{r}; Man^{+}	(20)
LGB6	GarB^{r}; Man^{+}	(20)
LGC1, LGC8, LGC13, LGC15	GarC^{r}; Man^{+}	(20)
PW202, PW203, PW204, LGN1, LGN2, LGN9	GarQ^{r}; Man^{+}	(21)
Lactococcus garvieae* IBB3403—other mutants*		
B548a	strain with *manABCD* deletion; Man^{-}	(20)
Lactococcus lactis* IL1403—spontaneous mutants*		
LLB2, LLB3, LLB17	RhmB^{r}; Man^{+}	This study
LLC1, LLC2	LcnC^{r}; Man^{+}	This study
LLG1-LLG3, LLG7-LLG9, LLG11, LLG12, LLG14, LLG19, LLG21, LLG25, LLG32, LLG46, LLG50	EntG1^{r}; Man^{+}	This study
LLA1, LLA7, LLA8, LLA12, LLA19-LLA22, LLA24, LLA26-LLA30	MurA^{r}; Man^{+}	This study
LLN1	GarQ^{r}; Man^{+}	(21)
M6, M16, M19, M30	BacSJ^{r}; Man^{+}	(22)
Lactococcus lactis* IL1403—other mutants*		
B464	IL1403 strain with *ptnABCD* deletion; GarABCQ^{r}, BacSJ^{r}; Man^{-}	LMGT NMBU (20, 21, 23)
B488	B464 carrying pNZ9530	LMGT NMBU (20, 21, 23)

B520	B488 carrying pNZ8037; GarABCQ^r, BacSJ^r; Man$^-$	LMGT NMBU (20, 21, 23)
B515	B488 carrying pNZ8037 with *ptnABCD*; GarAB^r, GarCQ^s, BacSJ^s; Man$^+$	LMGT NMBU (20, 21, 23)
B538	B488 carrying pNZ8037 with *ptnC*; GarABCQ^r, BacSJ^r; Man$^-$	LMGT NMBU (20, 21, 23)
B541	B488 carrying pNZ8037 with *ptnD*; GarABCQ^r, BacSJ^r; Man$^-$	LMGT NMBU (20, 21, 23)
B529	B488 carrying pNZ8037 with *ptnCD*; GarAB^r, GarCQ^s, BacSJ^s; Man$^+$	LMGT NMBU (20, 21, 23)
B557a	B488 carrying pNZ8037 with *manABCD*; GarABCQ^s, BacSJ^r; Man$^+$	(20)
B558a	B488 carrying pNZ8037 with *manCD*; GarABCQ^s, BacSJ^r; Man$^+$	(20)
B559a	B488 carrying pNZ8037 with *manC*; GarABCQ^r, BacSJ^r; Man$^-$	(20)
B560a	B488 carrying pNZ8037 with *manD*; GarABCQ^r, BacSJ^r; Man$^-$	(20)
B570a	B529 with Ser55Val substitution in *ptnC*; Man$^+$	This study
B571a	B529 with Ile59Phe substitution in *ptnC*; Man$^+$	This study
B572a	B529 with Val101Phe substitution in *ptnC*; Man$^+$	This study
B573a	B529 with Ile105Phe substitution in *ptnC*; Man$^+$	This study
B574a	B529 with Tyr198Val substitution in *ptnC*; Man$^+$	This study
B575a	B529 with Pro115Val substitution in *ptnD*; Man$^+$	This study
B576a	B529 with Thr134Val substitution in *ptnD*; Man$^+$	This study
B577a	B529 with Trp201Val substitution in *ptnD*; Man$^+$	This study
***Escherichia coli* EC1000**		
B529a	strain with pNZ8037 harboring *ptnCD*	(21)
B570a	B529a with Ser55Val substitution in *ptnC*; Man$^+$	This study
B571a	B529a with Ile59Phe substitution in *ptnC*; Man$^+$	This study
B572a	B529a with Val101Phe substitution in *ptnC*; Man$^+$	This study
B573a	B529a with Ile105Phe substitution in *ptnC*; Man$^+$	This study
B574a	B529a with Tyr198Val substitution in *ptnC*; Man$^+$	This study
B575a	B529a with Pro115Val substitution in *ptnD*; Man$^+$	This study
B576a	B529a with Thr134Val substitution in *ptnD*; Man$^+$	This study
B577a	B529a with Trp201Val substitution in *ptnD*; Man$^+$	This study
**Plasmids**		
pNZ9530	Em^r, carrying nisin-regulatory *nisRK* genes	(24)
pNZ8037	Cam^r, nisin regulated expression system with nisin-responsive promoter	(25)
**Primers**	Nucleotide sequence (5'->3')	
*ptnC*for/rev	TCTGACCTCTTTGGTTTG/GCATACGTTCGTAGT	
*ptnD*for/rev	AACCTCCAAGCTTCTG/AGCCACAGATTCTCTCC	
*manC*for/rev	CGTGATCTCGGCGTTA/TAACGCTCAAGCGTGTG	
*manD*for/rev	CGCTCTTATCTACCTC/GCCAATTTAGTGCTCCTAAC	
pNZ8037for/rev	CGATAACGCGAGCATA/GCTCAAGGGCTTTTACG	
*ptnC*Ser55Valfor/rev	GCAGGGATTATCCTCGGTGGTGTTCTTCAATTGATCGCTCTTGGT/ ACCAAGAGCGATCAATTGAAGAACACCACCGAGGATAATCCCTGC	
*ptnC*Ile59Phefor/rev	CGGTGGTTCACTTCAATTGTTTGCTCTTGGTTGGGCTAACG/ CGTTAGCCCAACCAAGAGCAAACAATTGAAGTGAACCACCG	
*ptnC*Val101Phefor/rev	CACATCATGGGTACTATCTTTCCTGCTGCTATCTTGC/ GCAAGATAGCAGCAGGAAAGATAGTACCCATGATGTG	
*ptnC*Ile105Phefor/rev	TACTATCGTTCCTGCTGCTTTTTTGCTTGCAACTGCTGGTC/ GACCAGCAGTTGCAAGCAAAAAAGCAGCAGGAACGATAGTA	
*ptnC*Tyr198Valfor/rev	TGGGATGGTTGTTGCCGTTGGTGTTGCAATGGTTATCAACC/ GGTTGATAACCATTGCAACACCAACGGCAACAACCATCCCA	
*ptnD*Pro115Valfor/rev	CTTGCCGGTATCGGTGACGTTGTCTTCTGGTTTACAGT/ ACTGTAAACCAGAAGACAACGTCACCGATACCGGCAAG	
*ptnD*Thr134Valfor/rev	TGGTGCGATTGCAGCTTCATTGGCTGTTGGTGGATCAATT/ AATTGATCCACCAACAGCCAATGAAGCTGCAATCGCACCA	
*ptnD*Trp201Valfor/rev	TTCGTCCTTGGTGTATTGATTCAACGTGTTGTAACAATTAACTTTAATGGTCCTAAC/ GTTAGGACCATTAAAGTTAATTGTTACAACACGTTGAATCAATACACCAAGGACGAA	
**Peptides**	Host organism	NCBI accession number(s)
GarQ	*Lactococcus garvieae* BCC 43578, plasmid	AEN79392.1
GarAG2	*Lactococcus garvieae* Lg-Granada, plasmid	WP_165719065.1; NZ_CP084378.1
LcnC/HB1	*Lactococcus cremoris* UC073	UXV60242.1
HB2	*Lactococcus garvieae* FDAARGOS_1002, plasmid	QQB43181.1
EntG1/HB3	*Enterococcus* sp. HSIEG1	EQC79567.1
HB4	*Ligilactobacillus agilis* 268A	PLA76810.1
AglA/HB5	*Ligilactobacillus agilis* UMB7763	MDK6810183.1

HB6	*Ligilactobacillus agilis* C7	MCL8204652.1
MurA/HB7	*Ligilactobacillus murinus* DSM 100193	MCR1897247.1
HB8	*Lactobacillus equicursoris* DSM 19284	CCK84999.1
HB9	*Furfurilactobacillus rossiae* DSM 15814	KRL52547.1
Bov255	*Streptococcus* sp. LRC 0255	AAG29818.1
Ubericin K	*Streptococcus uberis* LMGT 4214	QWX09803.1
Angicin	*Streptococcus anginosus* BSU 1211	UJH61018.1
HB10	*Leuconostoc carnosum* WC0328	KAA8327172.1
HB11	*Lacticaseibacillus paracasei* N53	MBU5324208.1
RhmB/HB12	*Lacticaseibacillus rhamnosus* VHProbi M14	UUT37922.1
HB13	*Lacticaseibacillus rhamnosus* TOM.283	MDM7524757.1
BacSJ	*Lacticaseibacillus paracasei* subsp. *paracasei* BGSJ2-8, plasmid	CAR92206.2
LcbC/HB14	*Companilactobacillus kimchii* DSM 13961	KAE9557329.1
HB15	*Lactococcus lactis* Bpl1	KGF75926.1
HB16	*Lactococcus lactis* 1031	MCT1192459.1
LcnA	*Lactococcus cremoris* 9B4, plasmid	P0A313.1
HB17	*Lactococcus lactis* FDAARGOS_866	WP_228777672.1, NZ_CP065734.1
LcnZ	*Lactococcus lactis* QU7	BAU29928.1
LcnB	*Lactococcus cremoris* 9B4, plasmid	P35518.1
HB18	*Lactococcus cremoris* TIFN3	EQC95783.1
HB19	*Lactobacillus* sp. CBA3605	AVK61836.1
HB20	*Lactococcus lactis* 537	MDG4972525.1
GarC	*Lactococcus garvieae* 21881, plasmid	CCF55362.1
LcnD/HB21	*Lactococcus* sp.	MBR6895708.1
HB22	*Enterococcus gallinarum* ENT.1	MBF0724730.1
MsnC/HB23	*Leuconostoc mesenteroides* 213M0	WP_061399677.1, NZ_BCMO01000030.1
HB24	*Leuconostoc mesenteroides* INF3b	MBZ1529843.1
HB25	*Leuconostoc mesenteroides* subsp. m*esenteroides* FM06, plasmid	ARR90014.1
HB26	*Leuconostoc citreum* LBAE C11	CCF27630.1
HB27	*Lactococcus lactis* 537	PFG87625.1
HB28	*Lactococcus* sp. EKM201L	KAF6605648.1
HB29	*Lactococcus cremoris* B40	KZK48428.1
HB30	*Lactococcus lactis*	MEE0824874.1
HB31	*Enterococcus faecalis* TX4248	EFM82101.1
GarA	*Lactococcus garvieae* 21881, plasmid	CCF71073.1
GarB	*Lactococcus garvieae* 21881, plasmid	CCF55365.1
GarAG3/HB32	*Lactococcus garvieae* M14, plasmid	CEF52411.1
GarAG1	*Lactococcus garvieae* Lg-Granada, plasmid	WP_225667055.1, NZ_CP084378.1

*Strains derive from the collections of: the Laboratory of Microbial Gene Technology, Department of Chemistry, Biotechnology and Food Science, Norwegian University of Life Sciences, Ås, Norway (LMGT NMBU); the Regional Strains and Plasmids Collection of the Institute of Biochemistry and Biophysics, Warsaw, Poland (IBB PAS); the Pure Cultures Collection of the Institute of Fermentation Technology and Microbiology, Technical University of Lodz, Poland (LOCK); the dietary supplement Dicoflor, Vitis Pharma, Poland (Dicoflor); the Collection of Microorganisms and Cell Cultures, Germany (DSMZ); the collection of the Dairy Institute of Asturias, Spanish National Research Council, Asturias, Spain (IPLA-CSIC); the collection of the National Institute for Agricultural Research, France (INRA), the American Type Culture Collection (ATCC), Centers for Disease Control and Prevention (CDC), otherwise they were obtained in earlier studies or in this study.

** Man – mannose phenotype (ability to grow on mannose as a sole carbon source)

Table S2. Sensitivity of *L. garvieae* IBB3403 and *L. lactis* IL1403 mutants to peptides with similarity to Man-PTS bacteriocins

Indicator strain	Genotype	MurA	MsnC	RhmB	AglA	LcbC	LcnC	LcnD	GarAG3	EntG1	HB10 HB13 HB15 HB19 HB20 HB27
L. garvieae											
IBB3403	wild-type	+	+/-	-	-	-	++	+	+	-	-
B548a	Δ*manABCD*	-	-	-	-	-	-	-	-	-	-
L. lactis											
IL1403	wild-type	+	+/-	+	+	+	++	+	-	+	-
B464	Δ*ptnABCD*	-	-	-	-	-	-	-	-	-	-
L. lactis Δ*ptnABCD*											
B520	pNZ8037	-	-	-	-	-	-	-	-	-	-
B557a	p*manABCD*	+	+/-	-	-	-	++	+	+	-	-
B558a	p*manCD*	+	+/-	-	-	-	++	+	+	-	-
B559a	p*manC*	-	-	-	-	-	-	-	-	-	-
B560a	p*manD*	-	-	-	-	-	-	-	-	-	-
B515	p*ptnABCD*	+	+/-	+	+	+	++	+	-	+	-
B529	p*ptnCD*	+	+/-	+	+	+	++	+	-	+	-
B538	p*ptnC*	-	-	-	-	-	-	-	-	-	-
B541	p*ptnD*	-	-	-	-	-	-	-	-	-	-

5 μl of each bacteriocin at a concentration of 1 mg/ml was used; "–", no inhibition zone (strain resistance); "+/–", minimal, vague inhibition zone (minimal strain sensitivity); "+" and "++", wide, clear inhibition zone with, respectively, diameter ≤ 10 mm and >10 mm (strain sensitivity)

Table S3. Spontaneous mutants of *L. lactis* IL1403 resistant to RhmB, EntG1, LcnC, MurA, GarQ, BacSJ, ubericin K, and GarC

Mutant strain	Mutation	Amino acid change	Sensitivity to [fold-decreased relative to WT]								Position in the cell membrane of affected Man-PTS amino acid(s)
			RhmB	EntG1	LcnC	MurA	GarQ	BacSJ	Ubericin K	GarC	
RhmB-resistant mutants											
LLB2,LLB3	*ptnD*: 376G>A	PtnD: Gly126Ser	8x	4x	0x	32x	0x	>4x	4x	0x	transmembrane (TH1)
LLB17	*ptnD*: 641C>T	PtnD: Ser214Leu	>16x	>8x	>128x	256x	256x	>4x	32x	0x	outside
EntG1-resistant mutants											
LLG1, LLG2	*ptnD*: 531-681multiple mutations	PtnD: 221-228del GlyAlaTyrLeuGluPheProLys	>16x	>8x	16x	>1024x	16x	>4x	>128x	>32x	outside (region γ)
LLG3, LLG9, LLG11, LLG32, LLG46	*ptnD*: 686G>T	PtnD: Gly229Val	>16x	>8x	16x	8x	16x	>4x	0x	>32x	outside (region γ)
LLG7, LLG19, LLG21	*ptnD*: 379G>A	PtnD: Ala127Thr	>16x	>8x	8x	16x	8x	>4x	32x	>32x	transmembrane (TH1)
LLG8	*ptnD*: 661G>T	PtnD: Gly221Cys	8x	>8x	16x	4x	16x	>4x	0x	>32x	outside
LLG12, LLG14	*ptnC*: 560C>T	PtnC: Ala187Val	0x	>8x	8x	32x	8x	>4x	2x	>32x	transmembrane (TH3)
LLG25	*ptnD*: 668A>G	PtnD: Tyr223Cys	>16x	>8x	32x	64x	16x	>4x	2x	>32x	outside (region γ)
LLG50	*ptnC*: 360G>T	PtnC: Met120Ile	8x	>8x	4x	64x	4x	>4x	8x	8x	transmembrane (TH1)
LcnC-resistant mutants											
LLC1, LLC2	*ptnC*: 185G> T	PtnC: Gly62Val	>16x	>8x	4x	128x	64x	>8x	64x	>32x	transmembrane
MurA-resistant mutants											
LLA1, LLA7, LLA8, LLA12, LLA19-LLA22, LLA24, LLA26-LLA30	*ptnC*: 328G>A	PtnC: Ala110Thr	4x	>8x	2x	>1024x	4x	>4x	64x	0x	transmembrane (TH1)
GarQ-resistant mutants											
LLN1	*ptnD*: 368C>A	PtnD: Pro123His	>16x	>8x	>128x	64x	>1024x	>8x	>128x	8x	transmembrane (TH1)
BacSJ-resistant mutants											
M6	*ptnD*: 599G>A	PtnD: Arg200His	8x	4x	2x	4x	8x	>8x	2x	>32x	transmembrane (TH3)
M16	*ptnD*: 247C>T	PtnD: Leu83Phe	8x	4x	2x	8x	0x	>8x	4x	4x	transmembrane (HP2b)
M19	*ptnD*: 677T>C	PtnD: Phe226Ser	>16x	>8x	8x	16x	8x	>8x	0x	>32x	outside (region γ)
M30	*ptnD*: 591G>T	PtnD: Leu197Phe	>16x	>8x	16x	32x	16x	>8x	4x	>32x	transmembrane (TH3)

“>” substitution; “del”deletion

Table S4. Spontaneous mutants of *L. garvieae* IBB3403 resistant to GarAG3, GarA, GarB, GarC, GarQ, MurA, and ubericin K

Mutant strain	Mutation	Amino acid change	Sensitivity to [fold-decreased relative to WT]							Position in the cell membrane of affected Man-PTS amino acid(s)
			GarAG3	GarA	GarB	GarC	GarQ	MurA	Ubericin K	
			GarAG3-resistant mutants							
LG1, 3-5, 7, 8, 10, 14-17, 19-21, 24-27, 30, 31, 34, 35, 37, 38, 40	*manD*: 608G >T	ManD: Arg203Leu	>1024x	0x	>64x	>128x	0x	0x	4x	transmembrane (TH3)
LG2	*manC*: 178G>T	ManC: Gly60Cys	1024x	4x	>64x	>128x	32x	>16x	>32x	transmembrane
LG6	*manD*: 767G>T	ManD: Trp256Leu	128x	0x	4x	8x	16x	0.5x	4x	extracellular (region γ+)
LG9	*manD*: 800G>T	ManD: Gly267Val	16x	0x	8x	2x	4x	0.5x	4x	extracellular (region γ+)
LG11, 13, 22, 28	*manD*: 607C>T	ManD: Arg203Cys	>1024x	0x	>64x	>128x	2x	0x	2x	transmembrane (TH3)
LG12	*manD*: 788_799del TTGTTGGTAACG	ManD: 262_265delAsnValValGly	64x	2x	>64x	8x	4x	0.25x	0x	extracellular (region γ+)
LG18	*manD*: 794_805del GTAACGGTGTTG	ManD: 265_268delGlyAsnGlyVal	32x	2x	8x	8x	4x	0.0625x	2x	extracellular (region γ+)
LG23, 29	*manC*: 563G>T	ManC: Gly188Val	>1024x	2x	>64x	>128x	4x	0x	8x	transmembrane (TH3)
LG32	*manD*: 953C>T	ManD: Ser318Leu	4x	2x	32x	>128x	2x	0x	>32x	extracellular
LG36	*manC*: 179G>T	ManC: Gly60Val	>1024x	8x	>64x	>128x	128x	>16x	>32x	transmembrane
			GarA-resistant mutants							
LGA2	*manC*: 176T>C	ManC: Leu59Pro	32x	32x	32x	32x	128x	4x	>32x	transmembrane (HP2a)
LGA3	*manC*: 155G>T	ManC: Gly52Val	32x	4x	64x	16x	4x	0.5x	8x	transmembrane (HP2a)
LGA6	*manC*: 570_571insGTT	ManC:190_191insVal	128x	8x	32x	64x	4x	0.5x	0x	transmembrane (TH3)
LGA10	*manD*: 173C>A	ManD: Ala58Asp	8x	2x	4x	4x	2x	2x	8x	transmembrane (HP2a)
			GarB-resistant mutant							
LGB6	*manD*: 937C>A	ManD: Gln313Lys	2x	0x	>64x	2x	2x	0.25x	4x	extracellular
			GarC-resistant mutants							
LGC1	*manD*: 102G>A	ManD: Met34Ile	8x	0x	0x	>128x	0x	0x	16x	transmembrane (HP1b)
LGC8	*manD*: 676T>G	ManD: Tyr226Asp	>1024x	8x	>64x	>128x	16x	0x	0x	extracellular (region γ)
LGC13	*manC*: 317C>T	ManC: Ala106Val	>1024x	2x	>64x	>128x	4x	4x	>32x	transmembrane (TH1)
LGC15	*manD*: 398C>T, 943G>A	ManD: Ala133Val, Val315Met	32x	2x	>64x	>128x	4x	0x	8x	transmembrane (TH1), extracellular
			GarQ-resistant mutants							
PW202	*manC*: 299C>A	ManC: Pro100His	2x	4x	0x	8x	>1024x	>16x	>32x	transmembrane (TH1)
PW203	*manD*: 368C>T	ManD: Thr123Ile	64x	0x	>64x	0x	>1024x	0x	>32x	transmembrane (TH1)
PW204	*manD*: 331C>T	ManD: Pro111Ser	64x	0x	>64x	0x	>1024x	>16x	>32x	transmembrane (TH1)
LGN1	*manD*: 902G>T	ManD: Gly301Val	256x	8x	>64x	32x	>1024x	2x	0x	extracellular (region γ)
LGN2	*manD*: 398C>T	ManD: Ala133Val	2x	0x	4x	16x	16x	2x	>32x	transmembrane (TH1)
LGN9	*manC*: 305C>T	ManC: Ala102Val	64x	0x	>64x	8x	16x	0.125x	0x	transmembrane (TH1)

5 µl of each bacteriocin at a concentration of 1 mg/ml was used; ">" substitution; "del"deletion; "ins" insertion

Table S5. ***L. lactis*** **B529 strains with site-specific mutations resistant to selected bacteriocins**

Mutant strain (Amino acid change)	Diameter of the inhibition zone [mm]												
	GarQ	BacSJ	GarC	Ubericin K	MurA	MsnC	Angicin	RhmB	AglA	LcbC	LcnC	LcnD	EntG1
B520	-	-	-	-	-	-	-	-	-	-	-	-	-
B529	24	12	+/-	16	9	+/-	7	+/-	9	7	19	6	+/-
B570a (Ser55Val in PtnC)	9	+/-	-	11	-	-	-	-	-	-	12	-	-
B571a (Ile59Phe in PtnC)	8	-	-	-	-	-	-	-	-	-	+/-	-	-
B572a (Val101Phe in PtnC)	10	-	-	+/-	+/-	-	-	-	-	-	19	-	-
B573a (Ile105Phe in PtnC)	10	-	-	+/-	+/-	-	-	-	7	-	13	6	-
B574a (Tyr198Val in PtnC)	9	-	-	10	+/-	-	-	-	-	-	12	-	-
B575a (Pro115Val in PtnD)	10	-	-	+/-	+/-	-	-	-	-	-	13	-	-
B576a (Thr134Val in PtnD)	10	-	-	12	+/-	-	-	-	+/-	-	14	-	-
B577a (Trp201Val in PtnD)	10	-	-	15	9	-	-	-	+/-	-	15	6	-

"–", no inhibition zone (strain resistance); "+/–", minimal, vague inhibition zone (minimal strain sensitivity)

Figure S1. Phylogenetic tree of peptides with similarity to Man-PTS-binding bacteriocins. The scale bar shows the evolutionary distance. Bootstrap values (%) are given in nodes.

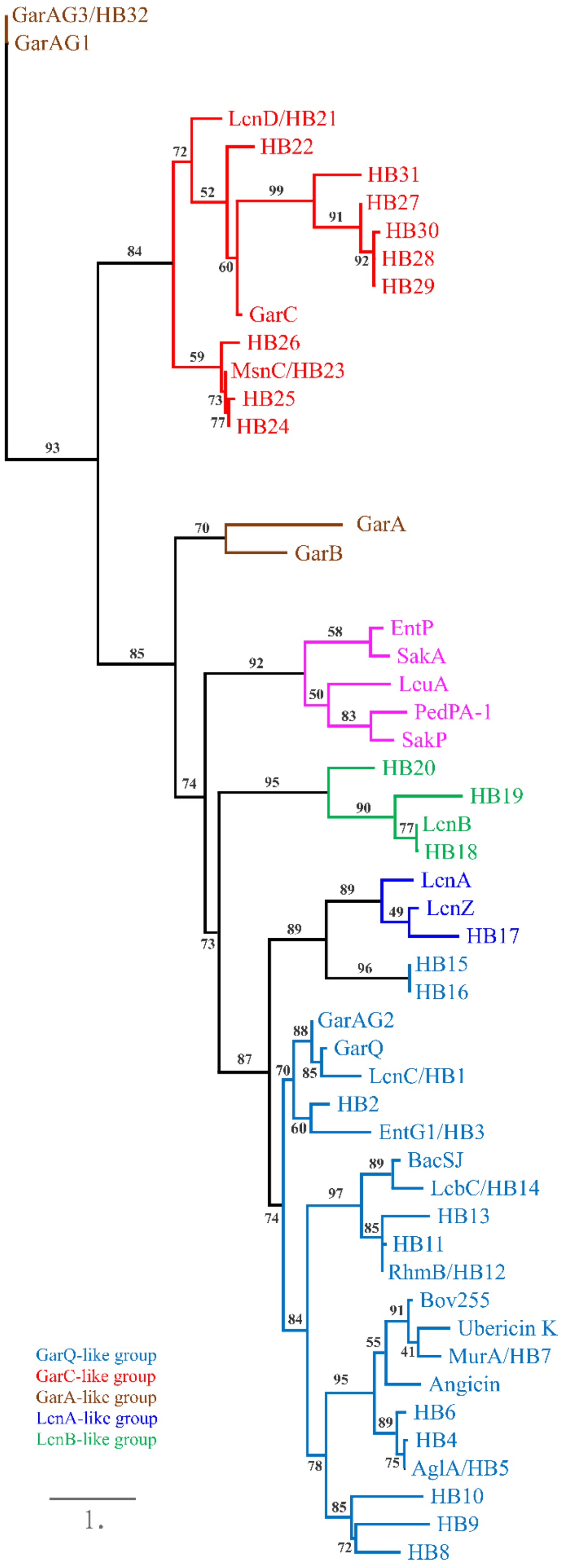

Figure S2. Tertiary structures of Man-PTS-binding bacteriocins. GarQ, GarA, GarC, and LcnB structures were predicted *in silico*, and PedPA-1 and LcnA structures were derived from PDB repository. The confidence in predicting tertiary structure is represented by a confidence score (C-score). The C-score typically ranges from -5 to 2, with a higher C-score indicating a more reliable model (26) . Localization of amino acid motifs conserved within the GarQ- and PedPA-1-like group is indicated.

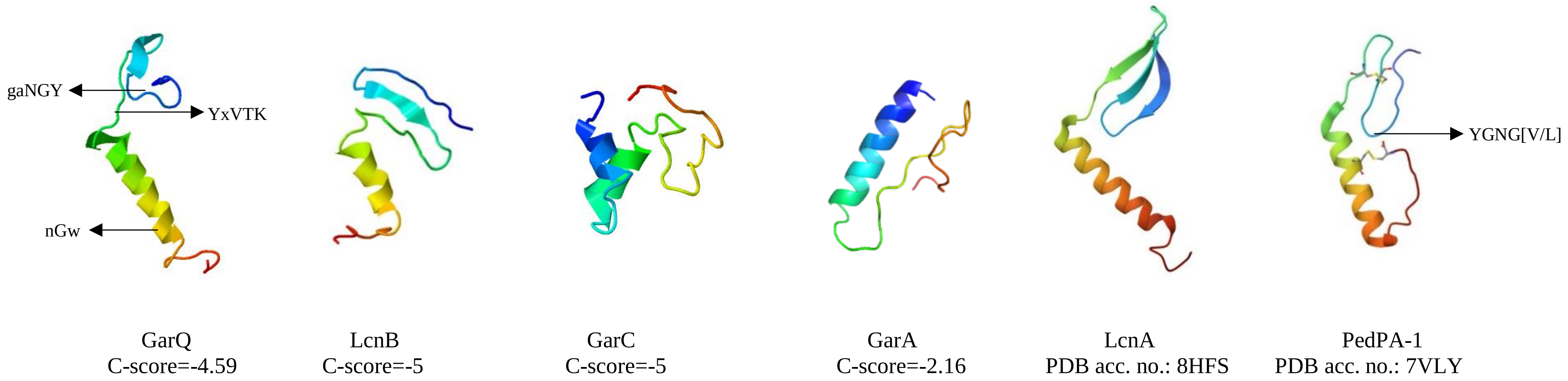

Figure S3. Inhibitory spectrum of peptides with similarity to Man-PTS-binding bacteriocins and of mutated variants of GarQ. Activity of GarQ, GarA, GarB, GarC, and BacSJ was partially taken from our earlier studies (20–22). Activity of LcnA and LcnB was predicted based on literature data (23). "no activity", no inhibition zone (strain resistance); "low activity", minimal, vague inhibition zone (minimal strain sensitivity); "medium activity" and "high activity", wide, clear inhibition zone with, respectively, diameter ≤ 10 mm and >10 mm (strain sensitivity). GarQ-, GarC-, GarA-, LcnA-, and LcnB-like bacteriocins are in blue, red, brown, navy, and green, respectively. The presence of Man-PTS region(s) α, γ, and γ+ is indicated in the third column. G− and G+ indicate Gram-negative and Gram-positive bacteria, respectively. *, does not contain the GG site of protease processing, thus no signal peptide was removed.

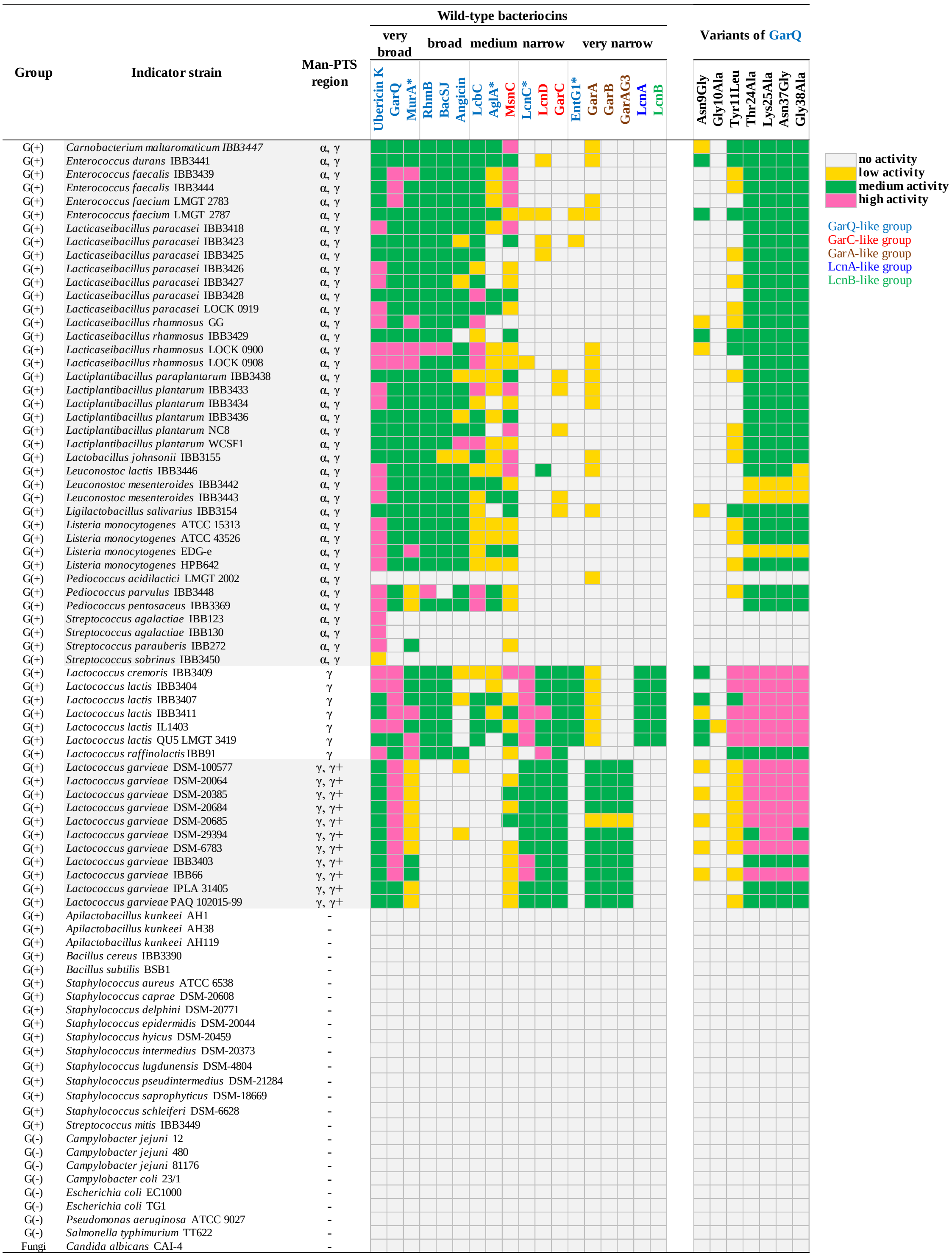

Figure S4. CD spectra of the HB10, HB13, HB15, HB19, HB20 and HB27 peptides with no antimicrobial activity. *, does not contain the GG site of protease processing, thus no signal peptide was removed.

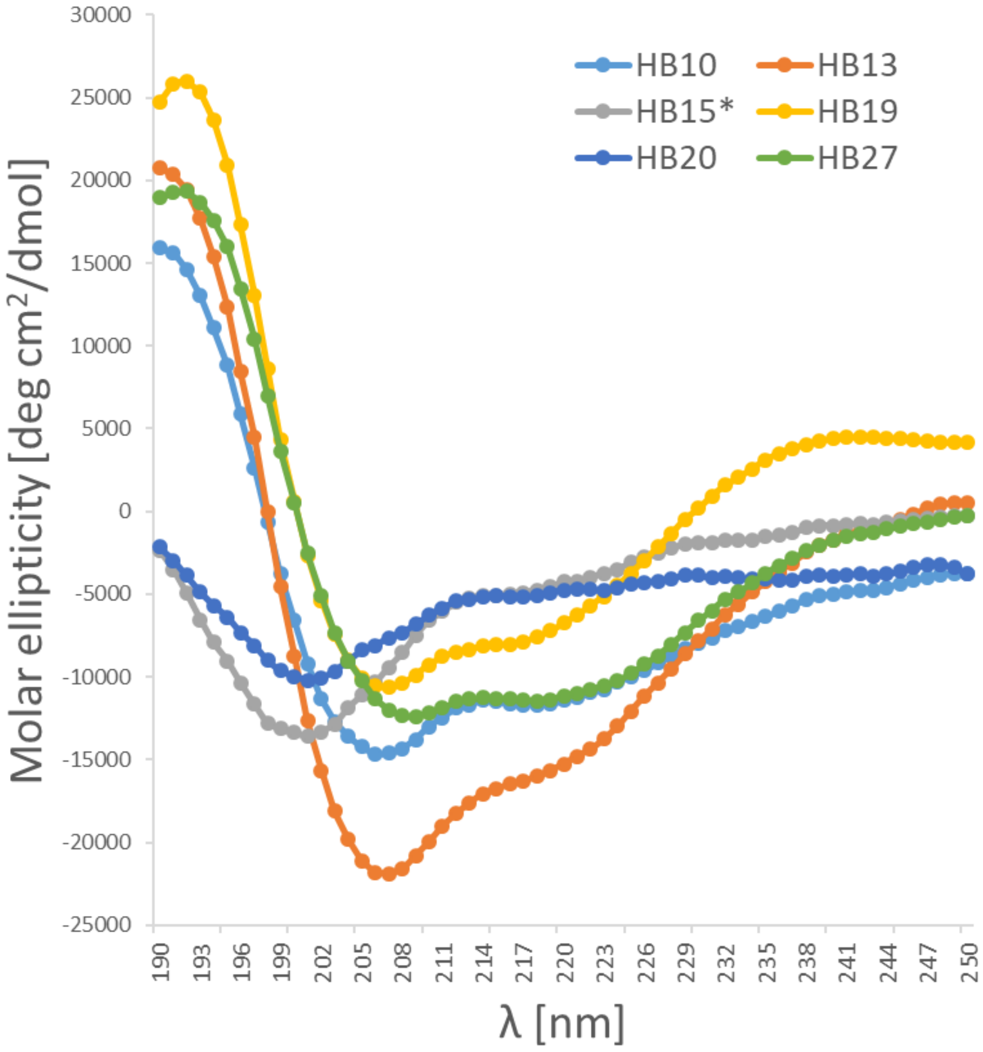

Figure S5. Sequence alignment of Man-PTS subunits IICIID from *E. coli*, *L. monocytogenes*, *L. lactis*, and *L. garvieae*. Asterisks, colons, and dots indicate fully, strongly and weakly conserved residues, respectively. Structural element symbols are: HP—hairpin, AH—amphipathic helix, TH—transmembrane helix. Within each subunit, coloured background was used to indicate: Core domain formed by HPs, TH1 and TH2—orange, Vmotif domain formed by TH3-TH5—blue, and AH—pink. The α, β, γ, and γ+ regions are underlined. The residues of Man-PTS$_{E. coli}$ involved in mannose or MccE492 binding are indicated by red or black background, respectively (27, 28). The residues of Man-PTS$_{L. monocytogenes}$ involved in interaction with PedPA-1 are indicated by grey background (29). The residues of Man-PTS$_{L. lactis/L. garvieae}$ affected by spontaneous or targeted mutations in bacteriocin-resistant mutants are indicated by green and navy blue background, respectively. Conserved residues changed by the mutations are in green boxes.

IIC

```
                  HP1a              HP1b           HP2a                      HP2b       region α          TH1
E. coli           MEITTLQIVLVFIVACIAGMGSILDEFQFHRPLIACTLVGIVLGDMKTGIIIGGTLEMIALGWMNIGAAVAPDAALASIISTILVIAGHQS-----IGAGIALAIPLAAAGQVLTIIVRTITV  118
L. monocytogenes  --MSVISIILVVLIAFLAGIEGILDEFQFHQPLIACTLIGLVTGNLTACIILGGTLQMIALGWANIGAAVAPDAALASVASAIILVLGGQGVAG-I-PSAIAIAIPLAVAGLFLTMIVRTLAV  119
L. lactis         MEYGVLSVILVIVVAFLAGLEGILDQWQFHQPIIACSLIGIVTGHASAGIILGGSLQLIALGWANVGAAVAPDAALASIASSILMVQSNNFDLTHIMGTIVPAAILLATAGLVLTTLVRMLSV  123
L. garvieae       --MSIISIILVLIFAFLAGLEGILDQWQFHQPIIACSLIGIATGHMAAGIILGGSLQMIALGWANVGAAVAPDAALASVASAILMVQGGNFDLTHITGVIVPAAILLATAGLVLTTLVRFLSV  121
                       :.::**.:.* :**: .***::***:*:***:*:*:. *.  : **:**:*::***** *:*************: *:*::: . :             :   ** **.** .** :** ::*

                                   TH2             AH           TH3                    TH4              TH5
E. coli           AFQHAADKAADNGNLTAISWIHVSSLFLQAMRVAIPAVIVALSVGTSEVQNMLNAIPEVVTNGLNIAGGMIVVVGYAMVINMMRAGYLMPFFYLGFVTAAFTNFNLVALGVIGTVMAVLYIQL  241
L. monocytogenes  PIVHLMDRAAEKGNIRSVEWLHISAICMQGIRIAIPAAAL-LFIPADSVQSFLEAMPAWLTDGMAIGGGMVVAVGYALVINMMATKEVWPFFVIGFVVAAISQLTLIAIGALGVALALIYLNL  241
L. lactis         VLVHQADRAAENGSYSGVEMWHFIALICQGLRIAIPAGLL-LVISPDAIQKALAAIPPVISGGLAVGGGMVVAVGYAMVINLMATREVWPFFFLGFALAPISELTLIATGVLGVVIAIVYLNL  245
L. garvieae       GIVHLADAAAEKGSYSGVAGWHMFALLLQGLRIAIPAAII-LAIPAETVTAALNAIPDWVSKGLAVGGGMVVVVGYAMVINLMATKELWPFFFLGFVLAPLSSITLIGMGILGVVIALIYLNL  243
                   : *   * **::*.  .:   *. ::   *.:*:****  : * :  . :    * *:*  :: *: :.***:*.****:***:*  :  : *** :**. * ::.:.*:. * :*..:*::*::*

                                region β
E. coli           SPKYNRVAGAPAQAAGNNDLDNELD--   266
L. monocytogenes  SKMGGGNSNGGGGGNSRDPLGDILNDY   268
L. lactis         QASGGSG--NGTASSSGDPIGDILNDY   270
L. garvieae       SNTAGNG---GTASSSGDPIGDILNDY   267
                  .   .         . . : :.: *:
```

IID

```
                            HP1a                   HP1b          HP2a               HP2b                  TH1
E. coli           MVDTTQTTTEKKLTQSDIRGVFLRSNLFQGSWNFERMQALGFCFSMVPAIRRLYPENNEARKQAIRRHLEFFNTQPFVAAPILGVTLALEEQRANGAEIDDGAINGIKVGLMGPLAGVGDPIF  123
L. monocytogenes  ------MAEKIELSKRDRLRVAWRSTFIQGSWNYERMQNGGWAFSMIPAIKKLYKTK-EDRSSALKRHLEFFNTHPYIASPILGVTLALEEERANGAEVDDVAIQGVKVGMMGPLAGVGDPVF  116
L. lactis         -----MSENKVTLDKKIRRSVMWRSMFLQGSWNYERMQNGGWAYSLIPALKKLYPSG-EEAKEALKRHLEFFNTHPYVAAPIIGVTLALEEERANGADIDDAAIQGVKVGMMGPLAGIGDPVF  117
L. garvieae       ---MENTNKKFSLSKNTRLSVMFRSMFLQGSWNYERMQNLGFLYSIIPALKQFYKPGSEEAKEALKRHMEFFNTHPYVAAPIVGVTLALEEEIANGVEIDEAAIQGVKVGMMGPLAGIGDPVF  120
                            :  * :     *  ** ::*****:****  *: :*::**::::*    *  ..*::**:*****:*::*:**:********: ***.::*: **:*:***:******:***:*

                                            TH2           AH       TH3
E. coli           WGTVRPVFAALGAGIAMSGSLLGPLLFFILFNLVRLATRYYGVAYGYSKGIDIVKDMGGGFLQKLTEGASILGLFVMGALVNKWTHVNIPLVV---SRITD----------------------  221
L. monocytogenes  WFTIRPMLGALGASLALSGNILGPILFFVAWNVIRWGFMWYTQEFGYKAGSKITDDLSGGLLQDITKGASILGMFVLAALVQRWVNIQFAP---IISKVKLDEGAYIDWSHLPQG--------  228
L. lactis         WFTVRPIVGAIAASLATGGSIIAPLFFFIVWNAIRIAFLWYTQEFGYKSGSAITKDLGGGLLQTVTKGASILGMFVLGVLIQRWVTINFNGPNAVVSKIPLQKGAYLEFP-------------  227
L. garvieae       WFTVRPIVGAIAASLATGGSIIAPIFFFVVWNAIRIAFLWYTQEFGYKQGTAITSDLGGGMLQQITKGASILGMFILGVLIQRWVNISFTGPNAMLPSKPLADGAYVGEWIDKAGKVVVQGAQ  243
                  * *:**:..*:.*.:* .*.::.*::**: :* :* :*  .  :*   :**. *  *..*:.**:** :*:******:*::..*:::*. ::

                                    region γ and region γ+                                         TH4              TH5
E. coli           -------------------------------------------------------QTGKEHVTTVQTILDQLMPGLVPLLLTFACMWLLRK---KVNPLWIIVGFFVIGIAGYACGLLGL  283
L. monocytogenes  ---------------------------------------------AQGIKTALEQQQAGLALSEIKVTTLQNNLDNLIPGLAAVALTFLCMWLLKK---KISPIIIILGLFVVGIVGHLIGLL--  303
L. lactis         ---------------------------------------KGSVSGTQLHDILGQVGNKLSLDPTKVTYLQDNLNQLIPGLAGLLITFLCMWLLKK---KVSPIVIIFGLFVVGILGRWAQIM--  307
L. garvieae       TGTTGDGVAKFDWLDQAGNVVGNGVAGQGGFAHYVTVDQLNTVDGSTLHNILGQVSSGLGLSPEQTQSLQDVFNSLIPGFIALLLTFLVLWILRKWKNKNAPLFIIIGMFVLGIVLHVAGLA--  365
                                                                      .   :.   :*   ::.*:**:  : :**  :*:*:*   *   *: **.*:**:**      :
```

Figure S6. Predicted structural elements of the membrane Man-PTS subunits IIC and IID of *L. lactis* IL1403 (A) and *L. garvieae* IBB3403 (B). Structural element symbols are: HP—hairpin, TH—transmembrane helix. HPs, TH1, and TH2 of IICIID subunits form the Core domain, and TH3-TH5 of IICIID subunits form the Vmotif domain. Spontaneous mutants obtained in the presence of GarQ, GarA, GarB, GarC, or BacSJ and their levels of resistance to these bacteriocins are from refs 20–22.

(A)

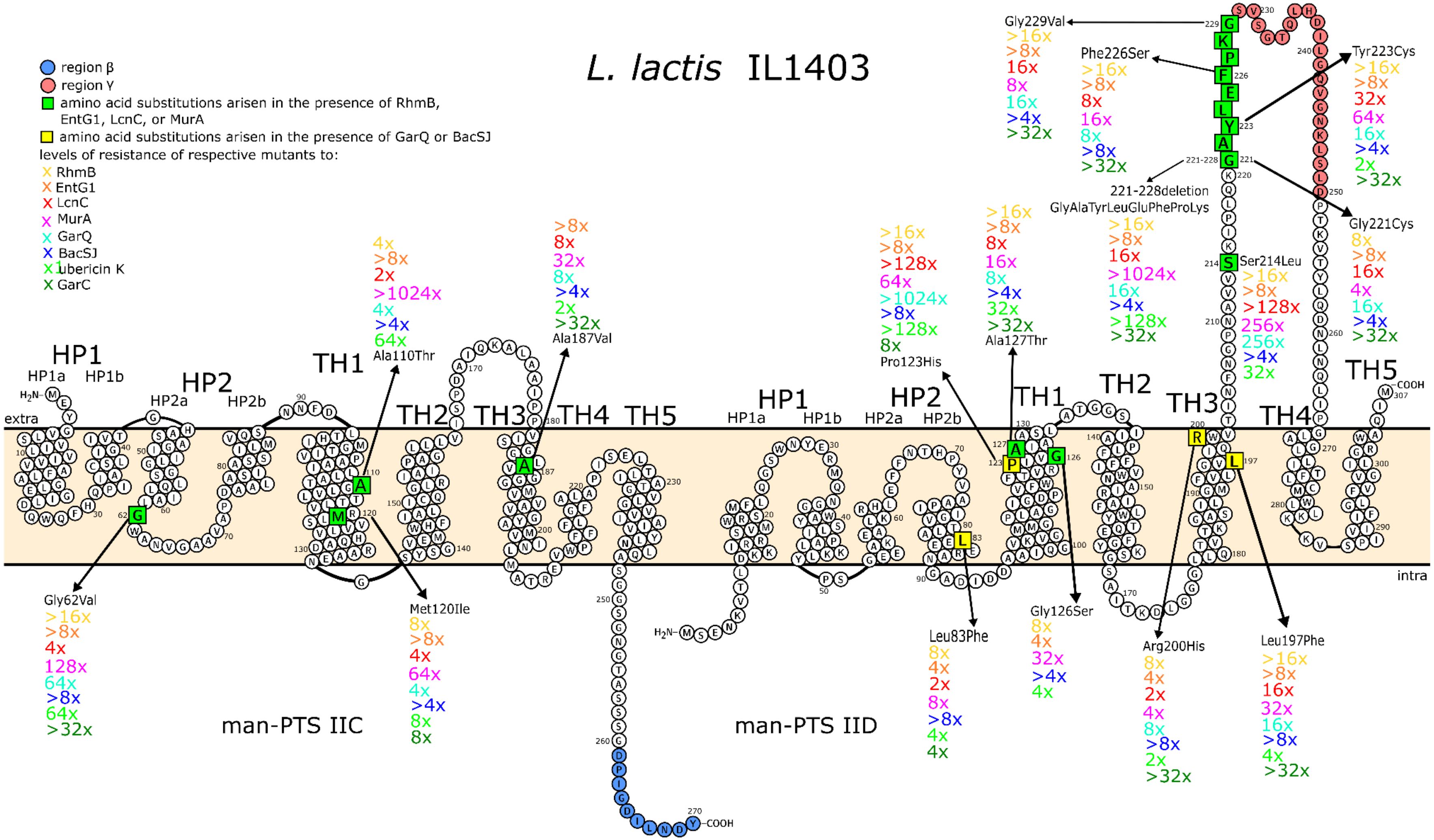

(B)

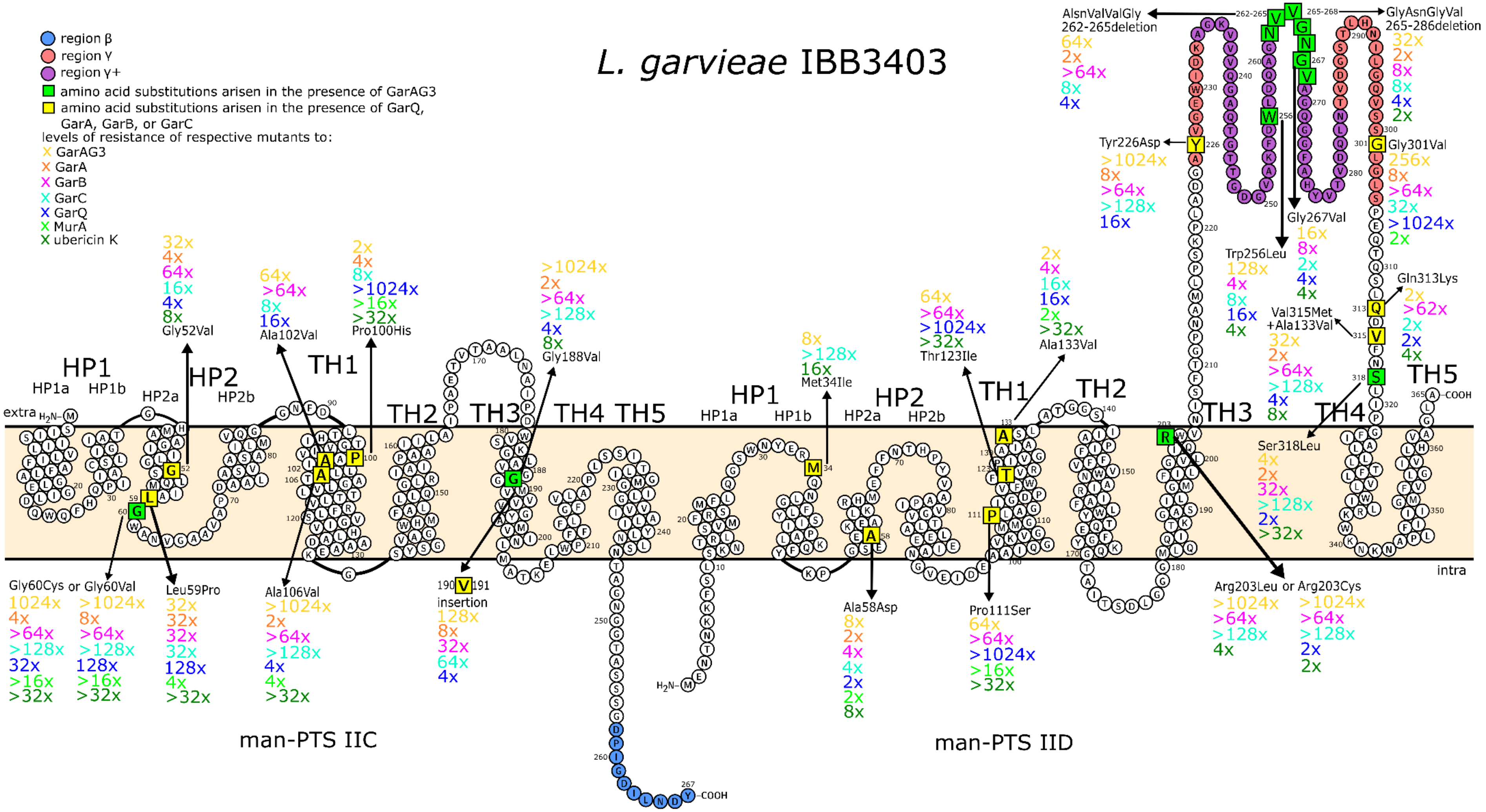

L. garvieae IBB3403
region β
region γ
region γ+
amino acid substitutions arisen in the presence of GarAG3
amino acid substitutions arisen in the presence of GarQ, GarA, GarB, or GarC
levels of resistance of respective mutants to:
X GarAG3
X GarA
X GarB
X GarC
X GarQ
X MurA
X ubericin K
extra
intra
HP1
HP1a
HP1b
HP2
HP2a
HP2b
TH1
TH2
TH3
TH4
TH5
man-PTS IIC
man-PTS IID
32x 4x 64x 16x 4x 8x Gly52Val
64x >64x 8x 16x Ala102Val
2x 4x 8x >1024x >16x >32x Pro100His
>1024x 2x >64x >128x 4x 8x Gly188Val
Gly60Cys or Gly60Val
1024x 4x >64x >128x 32x >16x >32x
>1024x 8x >64x >128x 128x >16x >32x
Leu59Pro 32x 32x 32x 32x 128x 4x >32x
Ala106Val >1024x 2x >64x >128x 4x 4x >32x
190 V 191 insertion 128x 8x 32x 64x 4x
8x >128x 16x Met34Ile
64x >64x >1024x >32x Thr123Ile
2x 4x 16x 16x 2x >32x Ala133Val
Ala58Asp 8x 2x 4x 4x 2x 2x 8x
Pro111Ser 64x >64x >1024x >16x >32x
Arg203Leu or Arg203Cys
>1024x >64x >128x 4x
>1024x >64x >128x 2x 2x
Ser318Leu 4x 2x 32x >128x 2x >32x
Val315Met +Ala133Val 32x 2x >64x >128x 4x 8x
Gln313Lys 2x >62x 2x 2x 4x
Trp256Leu 128x 4x 8x 16x 4x
Gly267Val 16x 8x 2x 4x 4x
Tyr226Asp >1024x 8x >64x >128x 16x
Gly301Val 256x 8x >64x 32x >1024x 2x
AlsnValValGly 262-265deletion 64x 2x >64x 8x 4x
GlyAsnGlyVal 265-286deletion 32x 2x 8x 8x 4x 2x
H2N–
–COOH

Figure S7. Sequence alignment of GarQ-like peptides active against *L. garvieae* and *L. lactis* (A) or *L. lactis* only (B). Fully conserved amino acids are in red, partially conserved ones—blue. Consensus symbols are: %—F or Y; !—I or V.

(A)

```
                    10        20        30        40        50        60        70
            ---------|---------|---------|---------|---------|---------|---------|-----
      GarQ                 EYHLMNGANGYLTRVNG--KYVYRVTKDPVSAVFGVISNGW-GSAGAGFGPQH
    GarAG2                 GTPLFYGANGYLTRENG--KYVYRVTKDPVSAVFGVISNGW-GSAGAGFGPQH
  LcnC/HB1  MVTTNKKSAPKKEPQFTKIMNSANGYLAYDNWNKKYVYHVTKDPVSAVAGVLANGW-GSAGAGFGPQTGGPSGKL
  MurA/HB7          MLKGKGYCKPVYYAANGYSCRYK-NGQWDYIVTKGNLEATLGVMSNGWVSSLGGGYFNRPK
Ubericin K             AKGVCKYVYPGSNGYACRYP-NGEWGYIVTKSNFEATKDVIVNGWVSSLGGGYFHGNRG
 Consensus  .....................gaNGYl.r.n.n.kyvY.VTKdpvsAv.gVisNGW.gSaGaG%gpq........
```

(B)

```
                    10        20        30        40        50        60        70
            ---------|---------|---------|---------|---------|---------|---------|--
    BacSJ                     YSYFGGSNGYSWRDKRGHWHYTVTKGGFETVIGIIGDGWGSAGAPGPGQH
 LcbC/HB14                     NFFGGSNGYSWRDKKGHWHYTVTSGVSSTVAQIIGNGWGSAGAPGVGQR
 RhmB/HB12                     VRIYGSNGYAWTDKHGHWHYTVTKGAVDTVFGIIGNGWGSAGASGRQH
  AglA/HB5  MAALLFFIKAAIRNSGYCEPVYYGANGYSCRYSNGKWDYKVTKTAAQAVGGVMAKGWIGSLGGSYYQPGYRG
   Angicin               GSGYCKPVMVGANGYACRYSNGRWDYKVTKGIFQATTDVIVKGW-AEYGPWIPRH
 EntG1/HB3          MRTIQGVG--RPIFNGANGYLSRDKYGHYTYTVTKGPLEAALGVMVNGRASTAGGGSWAPKYN
 Consensus  ...............g...p...GaNGY..rdk.Ghw.YtVTkg...av.g!i.nGw.s..g.g........
```

Figure S8. CD spectra of (A) GarQ in water, 25% TFE or 50% TFE, and (B) its amino acid variants in 50% TFE

(A) **(B)**

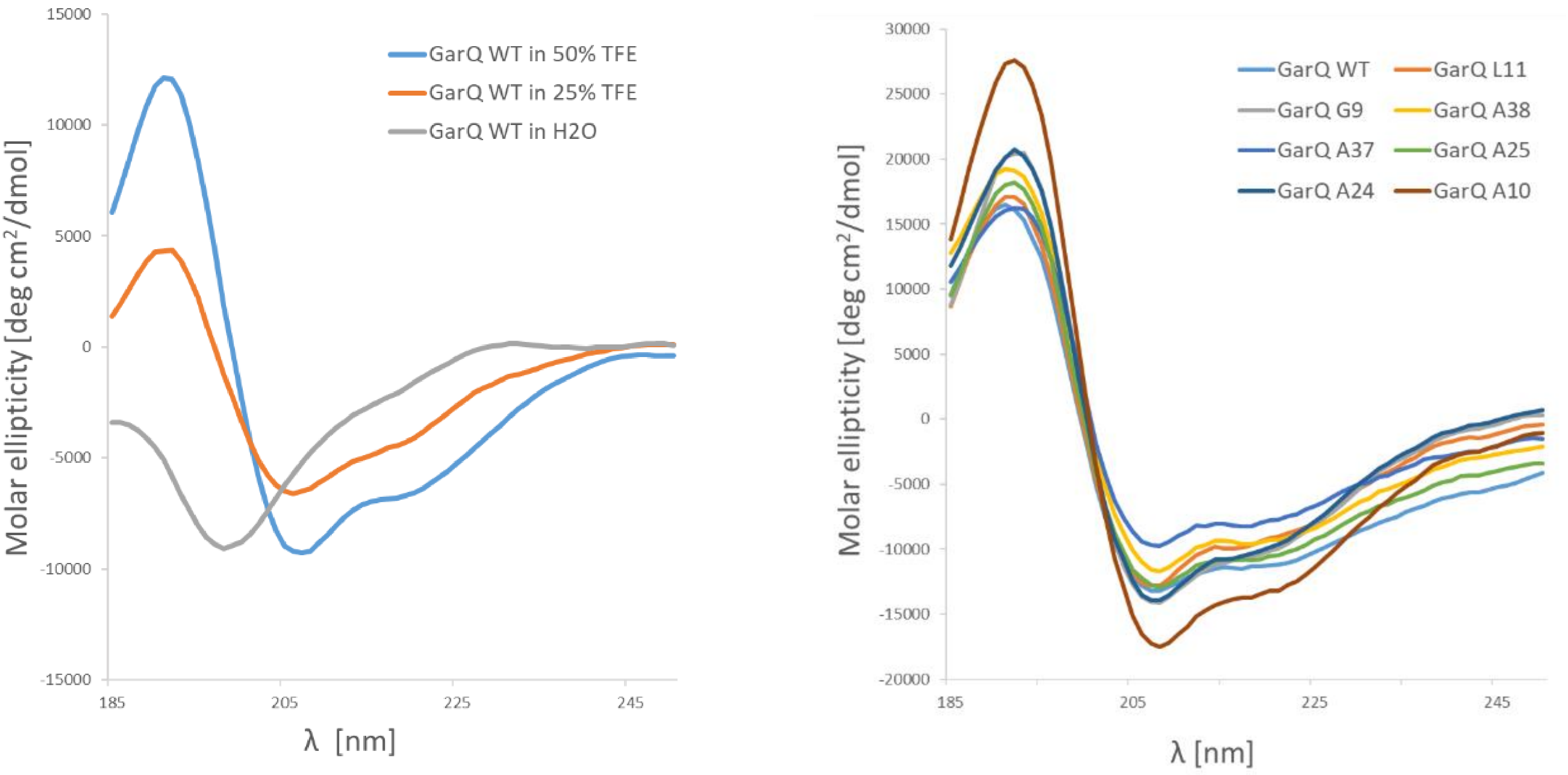

Figure S9. Schematic presentation of the arrangement of secondary structures in the main representatives of Man-PTS-binding bacteriocins. The proportions of the lengths of the peptides and their secondary structures have been preserved.

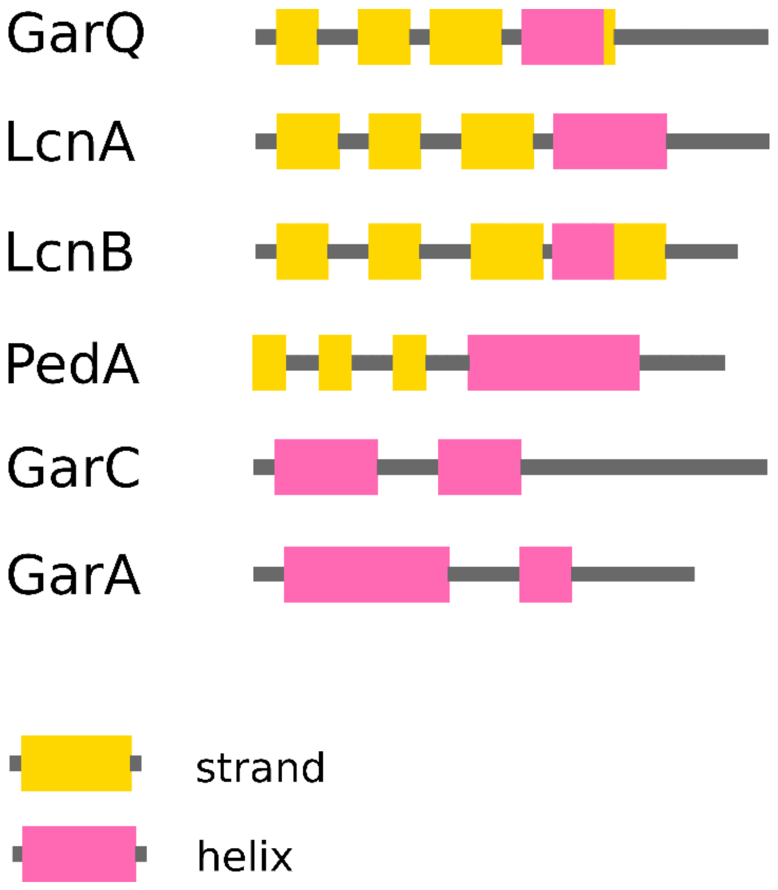

References

1. P. Nicolas, *et al.*, Condition-dependent transcriptome reveals high-level regulatory architecture in *Bacillus subtilis*. *Science* **335**, 1103–1106 (2012).
2. A. Wyszyńska, A. Raczko, M. Lis, E. K. Jagusztyn-Krynicka, Oral immunization of chickens with avirulent *Salmonella* vaccine strain carrying *C. jejuni* 72Dz/92 *cjaA* gene elicits specific humoral immune response associated with protection against challenge with wild-type *Campylobacter*. *Vaccine* **22**, 1379–1389 (2004).
3. A. D. Grabowska, *et al.*, *Campylobacter jejuni dsb* gene expression is regulated by iron in a Fur-dependent manner and by a translational coupling mechanism. *BMC Microbiol* **11**, 166 (2011).
4. J. A. Korlath, M. T. Osterholm, L. A. Judy, J. C. Forfang, R. A. Robinson, A point-source outbreak of campylobacteriosis associated with consumption of raw milk. *J. Infect. Dis.* **152**, 592–596 (1985).
5. W. A. Fonzi, M. Y. Irwin, Isogenic strain construction and gene mapping in *Candida albicans*. *Genetics* **134**, 717–728 (1993).
6. K. Leenhouts, *et al.*, A general system for generating unlabelled gene replacements in bacterial chromosomes. *Mol. Gen. Genet.* **253**, 217–224 (1996).
7. T. J. Gibson, Studies on the Epstein-Barr virus genome. (Cambridge University, 1984).
8. A. Koryszewska-Bagińska, T. Aleksandrzak-Piekarczyk, J. Bardowski, Complete genome sequence of the probiotic strain *Lactobacillus casei* (formerly *Lactobacillus paracasei*) LOCK919. *Genome Announc* **1** (2013).
9. A. Koryszewska-Bagińska, J. Gawor, A. Nowak, M. Grynberg, T. Aleksandrzak-Piekarczyk, Comparative genomics and functional analysis of a highly adhesive dairy *Lactobacillus paracasei* subsp. *paracasei* IBB3423 strain. *Appl. Microbiol. Biotechnol.* **103**, 7617–7634 (2019).
10. T. Aleksandrzak-Piekarczyk, A. Koryszewska-Baginska, J. Bardowski, Genome sequence of the probiotic strain *Lactobacillus rhamnosus* (formerly *Lactobacillus casei*) LOCK900. *Genome Announc* **1** (2013).
11. A. Koryszewska-Baginska, J. Bardowski, T. Aleksandrzak-Piekarczyk, Genome sequence of the probiotic strain *Lactobacillus rhamnosus* (formerly *Lactobacillus casei*) LOCK908. *Genome Announc* **2** (2014).
12. H. Morita, *et al.*, Complete genome sequence of the probiotic *Lactobacillus rhamnosus* ATCC 53103. *J. Bacteriol.* **191**, 7630–7631 (2009).
13. L. Axelsson, *et al.*, Genome sequence of the naturally plasmid-free *Lactobacillus plantarum* strain NC8 (CCUG 61730). *J. Bacteriol.* **194**, 2391–2392 (2012).
14. M. Kleerebezem, *et al.*, Complete genome sequence of *Lactobacillus plantarum* WCFS1. *Proc. Natl. Acad. Sci. U.S.A.* **100**, 1990–1995 (2003).
15. A. B. Flórez, *et al.*, Genome sequence of *Lactococcus garvieae* IPLA 31405, a bacteriocin-producing, tetracycline-resistant strain isolated from a raw-milk cheese. *J Bacteriol* **194**, 5118–5119 (2012).
16. M. C. Nelson, J. S. Varney, T. J. Welch, J. Graf, Draft genome sequence of *Lactococcus garvieae* strain PAQ102015-99, an outbreak strain isolated from a commercial trout farm in the northwestern United States. *Genome Announc* **4**, e00781-16 (2016).
17. A. Bolotin, *et al.*, The complete genome sequence of the lactic acid bacterium *Lactococcus lactis* ssp. *lactis* IL1403. *Genome Res.* **11**, 731–753 (2001).
18. P. Glaser, *et al.*, Comparative genomics of *Listeria* species. *Science* **294**, 849–852 (2001).
19. F. G. Chumley, R. Menzel, J. R. Roth, Hfr formation directed by Tn10. *Genetics* **91**, 639–655 (1979).
20. A. Tymoszewska, D. B. Diep, T. Aleksandrzak-Piekarczyk, The extracellular loop of Man-PTS subunit IID is responsible for the sensitivity of *Lactococcus garvieae* to garvicins A, B and C. *Sci Rep* **8**, 15790 (2018).
21. A. Tymoszewska, D. B. Diep, P. Wirtek, T. Aleksandrzak-Piekarczyk, The non-lantibiotic bacteriocin garvicin Q targets Man-PTS in a broad spectrum of sensitive bacterial genera. *Scientific Reports* **7**, 8359 (2017).
22. A. Tymoszewska, P. Walczak, T. Aleksandrzak-Piekarczyk, BacSJ—another bacteriocin with distinct spectrum of activity that targets Man-PTS. *International Journal of Molecular Sciences* **21**, 7860 (2020).
23. D. B. Diep, M. Skaugen, Z. Salehian, H. Holo, I. F. Nes, Common mechanisms of target cell recognition and immunity for class II bacteriocins. *PNAS* **104**, 2384–2389 (2007).

24. M. Kleerebezem, M. M. Beerthuyzen, E. E. Vaughan, W. M. de Vos, O. P. Kuipers, Controlled gene expression systems for lactic acid bacteria: transferable nisin-inducible expression cassettes for *Lactococcus*, *Leuconostoc*, and *Lactobacillus* spp. *Appl. Environ. Microbiol.* **63**, 4581–4584 (1997).
25. P. G. de Ruyter, O. P. Kuipers, W. M. de Vos, Controlled gene expression systems for *Lactococcus lactis* with the food-grade inducer nisin. *Appl Environ Microbiol* **62**, 3662–3667 (1996).
26. A. Roy, A. Kucukural, Y. Zhang, I-TASSER: a unified platform for automated protein structure and function prediction. *Nat Protoc* **5**, 725–738 (2010).
27. J.-M. Jeckelmann, B. Erni, The mannose phosphotransferase system (Man-PTS) - mannose transporter and receptor for bacteriocins and bacteriophages. *Biochim Biophys Acta Biomembr* **1862**, 183412 (2020).
28. K. Huang, J. Zeng, X. Liu, T. Jiang, J. Wang, Structure of the mannose phosphotransferase system (man-PTS) complexed with microcin E492, a pore-forming bacteriocin. *Cell Discov* **7**, 1–5 (2021).
29. L. Zhu, J. Zeng, C. Wang, J. Wang, Structural basis of pore formation in the mannose phosphotransferase system by pediocin PA-1. *Appl Environ Microbiol* **88**, e0199221 (2021).
